# Supplementary material for: A Rho Kinase (ROCK) Inhibitor, Y-27632, Inhibits the Dissociation-Induced Cell Death of Salivary Gland Stem Cells
Source: Molecules. 2021 May 1;26(9):2658. doi: 10.3390/molecules26092658 (PMC8124333; doi:10.3390/molecules26092658)
Supplement: Supplementary file 1 [file molecules-26-02658-s001.zip › molecules-1175745-supplementary.pdf]

Table S1. Primer sequences (5′ → 3′)

	Forward	Reverse
Aqp5	GCCCTCTTAATAGGCAACCAG	GCATTGACGGCCAGGTTAC
Mist1	GCTGACCGCCACCATACTTAC	TGTGTAGAGTAGCGTTGCAGG
Amy1	TCACACGGGTGATGTCAAGTT	GTCTGGGTTAATGCTCACTTCTT
Krt8	CAAGGTGGAACTAGAGTCCCG	CTCGTACTGGGCACGAACTTC
p53	GTCACAGCACATGACGGAGG	TCTTCCAGATGCTCGGGATAC
Bad	GAGGAGGAGCTTAGCCCTTT	AGGAACCCTCAAACTCATCG
Bcl2	GGTGGTGGAGGAACTCTTCA	ACCTACCCAGCCTCCGTTAT
Bax	AGACAGGGGCCTTTTTGCTAC	AATTCGCCGGAGACACTCG
Gapdh	AGGTCGGTGTGAACGGATTTG	TGTAGACCATGTAGTTGAGGTCA

Table S2. Primary antibodies

	Antibody	Dilution	Catalog number	Source
Primary antibodies	AQP5	1:500	bs-1554R	Bioss
	KRT8	1:500	bs-1106R	Bioss
	BCL-2	1:1000	NB100-56098	Novus Biologicals
	β-ACTIN	1:2000	GTX629630	GeneTex
Secondary antibodies	Mouse IgG	1:2000	GTX213111-01	GeneTex
	Rabbit IgG	1:2000	GTX213110-01	GeneTex
